# A web-based low carbohydrate diet intervention significantly improves glycaemic control in adults with type 2 diabetes: results of the T2Diet Study randomised controlled trial

**DOI:** 10.1038/s41387-023-00240-8

**Published:** 2023-08-27

**Authors:** Jedha Dening, Mohammadreza Mohebbi, Gavin Abbott, Elena S. George, Kylie Ball, Sheikh Mohammed Shariful Islam

**Affiliations:** 1 Institute for Physical Activity and Nutrition, School of Exercise and Nutrition Sciences, Deakin University, Burwood, VIC Australia; 2 Biostatistics Unit, Faculty of Health, Deakin University, Burwood, VIC Australia

**Keywords:** Patient education, Nutrition, Public health

## Abstract

**Background/objectives:**

In people with type 2 diabetes mellitus (T2DM), low carbohydrate diets (LCD), defined as 10–<26% total energy intake from carbohydrate, have indicated improved glycaemic control and clinical outcomes. Web-based interventions can help overcome significant challenges of accessibility and availability of dietary education and support for T2DM. No previous study had evaluated a web-based LCD intervention using a randomised controlled trial (RCT) design. The objective of this study was to assess whether a web-based LCD programme provided in conjunction with standard care improves glycaemic control in adults with T2DM.

**Subjects/methods:**

A 16-week parallel RCT was conducted remotely during Covid-19 among the general community, recruiting adults with T2DM not on insulin aged 40–89 years. Participants were randomly assigned (1:1) to standard care plus the web-based T2Diet healthy LCD education programme (intervention) or standard care only (control). The primary outcome was haemoglobin A1c (HbA1c). Secondary outcomes were weight, body mass index (BMI), anti-glycaemic medication, dietary intake, and self-efficacy. Blinded data analysis was conducted by intention-to-treat.

**Results:**

Ninety-eight participants were enrolled, assigning 49 to each group, with 87 participants (*n* = 40 intervention; *n* = 47 control) included in outcome analysis. At 16 weeks, there was a statistically significant between-group difference favouring the intervention group, with reductions in HbA1c –0.65% (95% CI: –0.99 to –0.30; *p* < 0.0001), weight –3.26 kg (*p* < 0.0001), BMI –1.11 kg/m^2^ (*p* < 0.0001), and anti-glycaemic medication requirements –0.40 (*p* < 0.0001), with large effect sizes Cohen’s *d* > 0.8.

**Conclusion:**

This study demonstrated that as an adjunct to standard care, the web-based T2Diet programme significantly improved glycaemic control and clinical outcomes in adults with T2DM. In addition, the results highlight the potential to improve access and availability for people with T2DM to achieve glycaemic control and improved health through web-based dietary education and support.

## Introduction

For people with type 2 diabetes mellitus (T2DM), dietary modification is essential, as it has been demonstrated to support successful achievement and maintenance of glycaemic targets and optimisation of health outcomes [1, 2]. Currently, for people with T2DM, there is no recommended ideal amount for any macronutrient [1, 2]. It has long been understood, however, that carbohydrate is the nutrient with greatest impact on glycaemic control. Thus, for decades, carbohydrate reduction and counting have been fundamental recommendations for improved glycaemic self-management in people with T2DM [2, 3]. High carbohydrate diets are defined as >45% carbohydrate from total energy intake, moderate carbohydrate diets 26–45%, low carbohydrate diets (LCDs) 10–<26%, and ketogenic diets <10% [4, 5]. A large body of evidence now exists to support carbohydrate reduction as one of the most effective strategies for improving glycaemic control and other clinical outcomes [6–13]. Both ketogenic diets and LCDs have indicated improved glycaemic control and reductions in body weight and medication requirements [6–10], although across reviews LCDs were shown to have greater dietary adherence [6, 7, 9, 10]. Historically, LCDs have been misunderstood as being unhealthy [14]. However, LCDs focus on the core elements recommended by the American Diabetes Association as fundamental to all healthy eating patterns in T2DM—high consumption of non-starchy vegetables and nutrient-dense foods, and low consumption of added sugar, refined grains, and processed foods [2, 14]. Importantly, LCDs are supported by international diabetes guidelines as a suitable dietary choice for people with T2DM [2, 15–19].

While dietary modification is essential, it is a task that people with T2DM need to self-manage. To do so, they often need dietary education and support [1, 2]. It is also recognised that T2DM self-management education and support is an ongoing requirement [20, 21]. However, there are significant challenges in reaching enough people with T2DM with the education and support they need [21–24]. This is due to the growing number of people with T2DM and lack of workforce capacity to meet the demand [21, 22, 24]; location—health services are limited in rural and remote areas [21, 25]; inadequately subsidised health reimbursement schemes [21, 26, 27]; and the time, labour, and cost involved in delivering face-to-face programmes and services [2, 21, 22, 24, 26, 28]. These barriers of access and availability were further exacerbated by coronavirus disease 2019 (Covid-19) [29]. Thus, improving access and availability of dietary education and support through other means is urgently needed [2, 21]. Provided as an adjunct to standard care from a GP or multidisciplinary team, web-based interventions show potential to overcome these barriers by providing an accessible means to facilitate positive health changes in people with T2DM, which in turn could have a substantial impact on the healthcare system [2, 30]. No previous study has assessed a web-based LCD intervention using an RCT design. The primary aim of this study was to evaluate the effectiveness of standard care plus a web-based healthy LCD education programme compared to standard care only on glycaemic control in adults with T2DM. Secondary aims were to evaluate intervention changes in anti-glycaemic medication, body weight, BMI, dietary intake, and self-efficacy between the two groups. We hypothesised that standard care plus web-based LCD programme would result in lower HbA1c levels at 16 weeks compared to standard care only.

## Subjects and methods

### Study design

This study employed a two-arm parallel randomised design, conducted remotely during the Covid-19 pandemic among the general community Australia-wide, including metropolitan, rural, regional, and remote areas. The study protocol with a detailed description of participants, interventions, and procedures was previously published [31]. Minor modifications during early recruitment included removal of two exclusion criteria and updated data collection methods. Ethics approval was obtained from Deakin University Human Research Ethics Committee (2020–349).

### Participants

Recruitment of participants occurred through community organisations, social media, collegial networking, and in the last few weeks of recruitment through paid Facebook advertising to bolster enrolments (*n* = 10). Participants were eligible to enrol if they had T2DM not on insulin with self-reported HbA1c levels ≥7.0% within six months of enrolment; 40–89 years of age; English-speaking; had internet access; an active email address; and based in Australia. Participants were excluded if they had other forms of diabetes; were vegan or vegetarian; had bariatric surgery; diagnosed cardiovascular or renal disease; a condition affecting their ability to participate; were pregnant or lactating; on a weight loss programme or had taken a weight loss programme within three months of enrolment; were enrolled in other clinical studies; at risk of disordered eating screened with the Eating Attitudes Test-26 [32]; or baseline HbA1c measurement was returned as non-diabetic ≤5.6%. Written informed consent was collected from all participants.

### Randomisation and blinding

Participants were randomly assigned (1:1) to standard care plus the web-based T2Diet healthy LCD education programme (intervention) or standard care only (control). The computer-generated random allocation sequence used random varying block sizes of 2 and 4, and was held off-site by a statistician. Randomisation was stratified by gender and age (strata 40–60 years and 61+ years). Once eligible, participants were contacted by email to complete baseline measures. Once completed, participant study ID and stratification factors were logged onto a secure remote spreadsheet. The statistician recorded the logs before indicating the group allocations. Participants were then notified by email of their group allocation from the lead researcher.

Researchers and participants were blinded to group allocation until after group assignment. Assessment of the primary outcome was blinded, as the pathology lab had no disclosure of group allocation. Secondary outcomes were assessed via participant self-report, for both groups. Where there was unclear or incomplete data, a research assistant blinded to group allocation clarified dietary intake data collected. A statistician cleaned and collated the data set in preparation for analysis. The blinded dataset was provided to a separate study statistician to perform blinded data analysis.

### Procedures

The intervention was detailed in the published protocol [31]. In brief, following completion of baseline measures, intervention group participants were sent an email with login details to access the web-based T2Diet programme. The intervention was an automated 16-week healthy LCD education programme. Web-based recommendations encouraged carbohydrate intake of 50–100 g per day with ad libitum consumption of nutrient-dense lower carb foods, emphasising high intake of non-starchy vegetables and dietary fibre, and decreased intake of starches, sugar, and discretionary foods. There were no specific prescriptions for protein and fat intake, though nutrient-dense sources were emphasised. Approximately three days after provision of login details, intervention group participants were contacted by phone or email to discuss potential adverse effects of carbohydrate reduction and cautions regarding anti-glycaemic medications [31]. During the contact, intervention group participants were instructed to report any adverse effects via an online form or by contacting the lead researcher; to continue with their standard care; and encouraged to communicate their participation in the study with their GP. Intervention group participants were then advised to login at least once per week and left to participate in the automated programme.

Following completion of baseline measures, participants in the control group were instructed to continue with their standard care. At 16 weeks, participants in both groups were contacted via email to complete end-of-study measures.

### Outcomes

The primary outcome measure was HbA1c (Nutripath Integrative Pathology Service, Victoria, Australia). Participants were sent Nutripath HbA1c test kits in the mail, which they returned by mail to the pathology centre for assessment. Self-reported secondary outcomes were changes in anti-glycaemic medications, quantified based on potency and dosage using the Medication Effect Score (MES) [33]; dietary intake assessed via 24-hour recall and analysed using FoodWorks Professional (Xyris Pty Ltd, Brisbane, v10.0); self-efficacy measured via the Diabetes Management Self-Efficacy Scale—Australian version (DMSES) [34]; and weight and BMI (kg/m^2^) based on self-reported height and weight. A checklist of diabetes-related comorbidities was included as an exploratory outcome. Demographics and height were collected at baseline. All other measures were collected at baseline, prior to randomisation, and 16 weeks later. No change to trial outcomes occurred after trial commencement.

### Statistical analysis

A sample size of 100 participants (50 per group) provided 80% power with a 5% two-sided *a* to detect a between-group difference in HbA1c of 0.5% at 16 weeks [31]. The sample size assumed a standard deviation of 0.9 HbA1c, a pre-post intervention correlation of 0.5, and a 20% dropout. Intervention effects (i.e., mean differential change of HbA1c from baseline at week 16 between intervention and control group) were estimated using a generalised estimation equation (GEE) model for continuous outcomes assuming Gaussian distribution with an identity link. The GEE model included HbA1c measure as the dependent variable, nominal treatment group and measurement time (i.e., baseline and week 16) and the two-way interaction between group allocation and time as independent variables, while adjusting for stratification variables (age and gender). The two-way interaction between group allocation and time estimated the intervention effect as differential change of HbA1c at baseline between the intervention and control group at week 16. Multivariate multiple imputation using chained equations with group allocation, age, gender, time-updating BMI, and conditional mean of previous time-point HbA1c measure as auxiliary variables [35] was used to impute missing data to comply with the intention-to-treat approach (number of imputations=50). All other continuous secondary outcomes were analysed in a similar manner. All hypothesis tests were two-sided, and P value < 0.05 was considered as the level of significance for the primary outcome and all secondary outcomes. Cohen’s *d* effect size was reported, with 0.2 representing a small effect size, 0.5 a medium effect size, and 0.8 a large effect size [36]. Stata version 17 was used for quantitative analysis. This trial is registered with the Australian New Zealand Clinical Trials Registry, number ACTRN12621000096853.

## Results

**Fig. 1:**
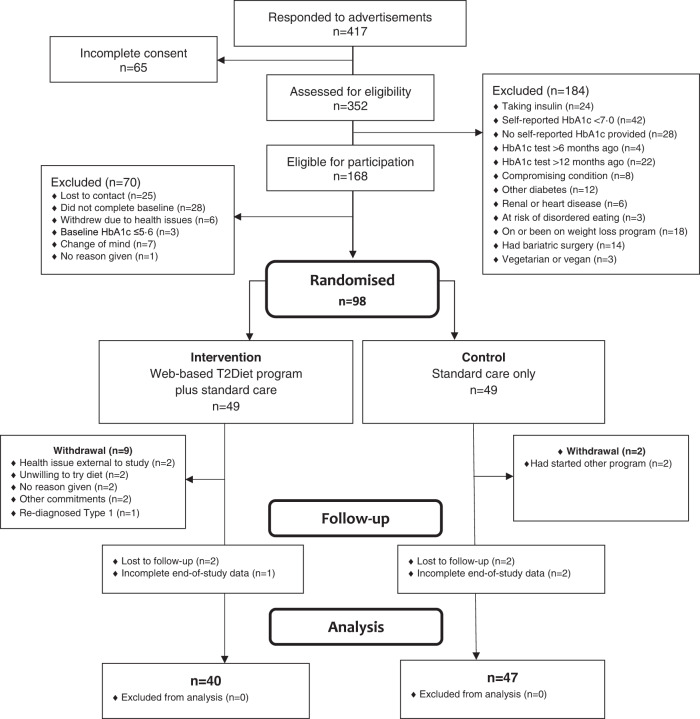
CONSORT flowchart of the study. Overview of the 16-week parrallel randomised trial from recruitment through to analysis.

Recruitment took place between Feb 1, 2021 and Oct 10, 2021. Follow-up was completed on Feb 18, 2022. Of 352 screened for eligibility, a total of 98 participants were enrolled into the study, with 49 participants allocated to each group (Fig. 1). Eleven participants withdrew consent from participation in the study and use of their data. Eighty-seven participants were included in outcome analysis; 40 intervention group participants (82%) and 47 control group participants (96%). Seven missing primary outcome values were imputed during intention-to-treat analysis, as two participants from each group were lost to follow up, and one participant in the intervention group and two in the control group failed to complete end-of-study data.

Baseline characteristics were similar between groups (Table 1). For the whole study population at trial entry, mean age was 60.5 years (SD 9.5), and mean clinical values were HbA1c 7.7% (SD 1.2), body weight 100.07 kg (SD 21.74), and BMI 34.73 kg/m^2^ (SD 7.25). Eighty-five per cent of participants (*n* = 74) were taking anti-glycaemic medication.

**Table 1:** Demographic characteristics of the participants.

Demographic category	Demographic details	Intervention group (*n* = 40)	Control group (*n* = 47)
Gender, *n* (%)	Female	23 (57)	29 (62)
	Male	17 (43)	18 (38)
Age (years), mean (SD)		61.3 (9.4)	59.8 (9.6)
Duration of T2DM, *n* (%)	<1 year	8 (20)	7 (15)
	1–6 years	14 (36)	18 (38)
	7–15 years	14 (36)	12 (26)
	>15 years	4 (10)	10 (21)
Family history of T2DM, *n* (%)	Yes	19 (47.5)	22 (46.8)
	No	21 (52.5)	25 (53.2)
Country of birth, *n* (%)	Australia	31 (78)	35 (74)
	International	9 (24)	12 (25)
Relationship status, *n* (%)	Married/living with a partner	31 (78)	35 (74)
	Separated, divorced, widowed	7 (18)	10 (21)
	Never married	2 (5)	2 (4)
Education level, *n* (%)	Bachelor’s degree or above	13 (33)	13 (27)
	Tertiary level/trade certificate	13 (33)	24 (51)
	Completed high school	9 (23)	7 (15)
	None	5 (13)	3 (6)
Employment status, *n* (%)	Employed full time	10 (25)	19 (40)
	Employed part time	7 (18)	7 (15)
	Retired	17 (43)	16 (34)
	Unemployed	6 (17)	5 (10)
Smoker, *n* (%)	Yes	1 (2.5)	2 (4.3)
	No	39 (97.5)	45 (95.7)
Anti-glycaemic medication, *n* (%)	No	10 (25)	3 (6)
	Yes	30 (75)	44 (94)
Medication class, *n* (%)	Metformin	28 (70)	41 (87)
	Sulfonylureas	10 (25)	6 (13)
	DPP-4 inhibitors	10 (25)	16 (34)
	GLP-1 agonists	5 (13)	5 (11)
	SGLT-2 inhibitors	8 (20)	15 (32)

**Fig. 2:**
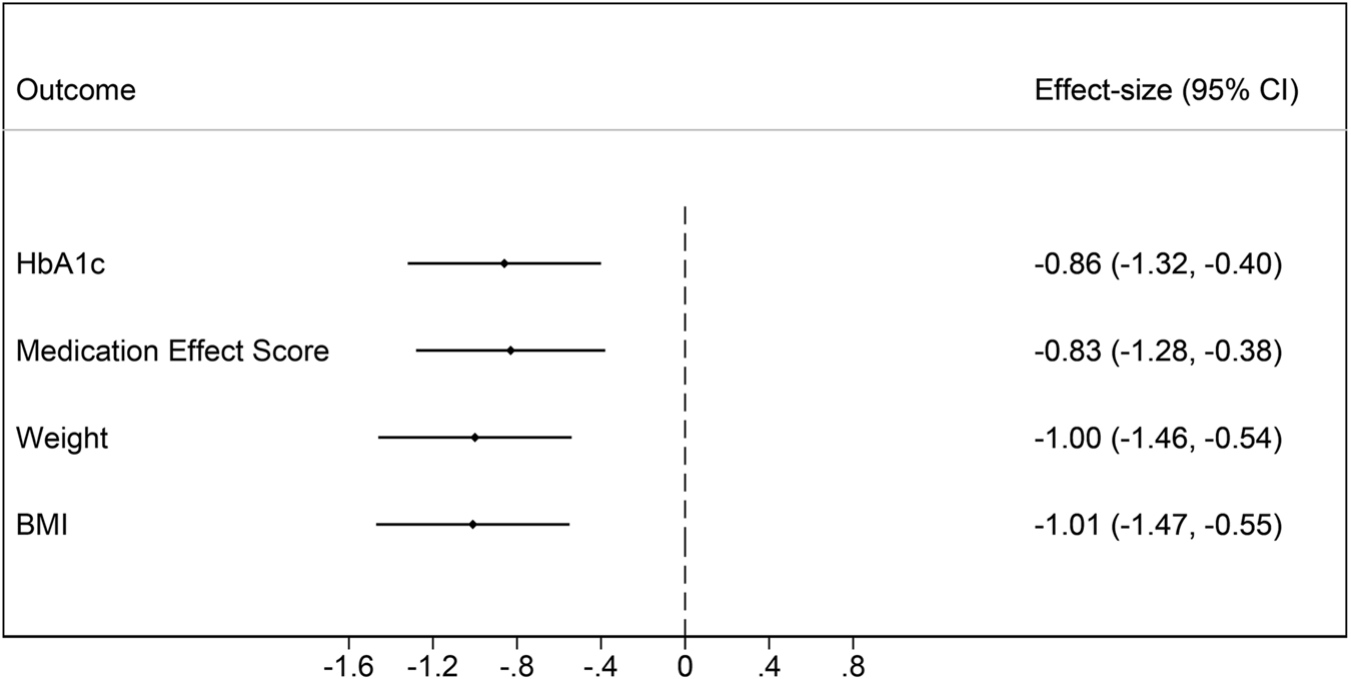
Cohen’s *d* effect size for primary and secondary clinical outcomes. For Cohen’s *d*, 0.2 represents a small effect size, 0.5 a medium effect size, and 0.8 a large effect size.

For the primary outcome, at 16 weeks, the intention-to-treat analysis showed a statistically significant between-group mean differential change (*p* < 0.0001), with intervention group participants achieving greater reductions in HbA1c (Table 2). For secondary clinical outcomes, there was a statistically significant between-group mean differential change in weight (*p* < 0.0001), BMI (*p* < 0.0001), and anti-glycaemic medication (*p* < 0.0001), favouring the intervention group (Table 2). Thirty-eight per cent (*n* = 14) of intervention group participants lost ≥5% body weight, compared to 9% (*n* = 3) of control group participants. Anti-glycaemic medication requirements were reduced in the intervention group and increased in the control group. Twenty-five per cent (*n* = 7) of the intervention group had ≥20% medication reduction. The primary and secondary clinical outcomes all had large effect sizes (Fig. 2).

**Table 2:** Primary and secondary clinical outcomes at baseline and 16 weeks of the two groups and intervention effect.

Outcome	*n*^a^	Mean (SD)			Intervention effect^b^	
		Baseline	16 weeks	Within-group change	Estimate (95% CI)	*p* value
HbA1c, %						
Intervention	37	7.64 (1.24)	6.79 (1.14)	−0.94 (0.86)	−0.65 (−0.99, −0.30)	<0.0001
Control	43	7.89 (1.30)	7.57 (1.28)	−0.26 (0.72)		
MES						
Intervention	38	1.46 (1.30)	1.31 (1.17)	−0.09 (0.56)	−0.40 (−0.62, −0.19)	<0.0001
Control	45	1.47 (0.87)	1.80 (0.92)	0.34 (0.49)		
Weight, kg						
Intervention	37	98.30 (19.29)	92.72 (18.84)	−4.36 (3.66)	−3.26 (−4.81, −1.71)	<0.0001
Control	45	101.57 (23.72)	100.74 (23.09)	−0.77 (3.52)		
BMI						
Intervention	37	33.89 (6.16)	31.99 (6.13)	−1.48 (1.19)	−1.11 (−1.63, −0.59)	<0.0001
Control	45	35.44 (8.07)	35.11 (7.92)	−0.27 (1.20)		

*BMI* body mass index, *CI* confidence interval, *HbA1c* haemoglobin A1c, *MES* Medication Effect Score, *SD* standard deviation.

^a^Number of participants with data available at 16 weeks for each outcome. All participants had complete data at baseline (*n* = 40 intervention group, *n* = 47 control group).

^b^Between-group differential change from baseline to week 16 (intervention versus control) estimated through two-way interaction between group allocation and measurement time point.

Table 3 presents the mean dietary intake and percentages for both groups. At 16 weeks, between-group differential change in carbohydrate intake was significant (*p* < 0.0005). There were no significant differences between groups in terms of energy intake, protein, saturated fat, or dietary fibre. There was a significant between-group difference for total fat intake (*p* = 0.040). The intervention group significantly increased monounsaturated (*p* = 0.034) and polyunsaturated fat intake (*p* = 0.003).

**Table 3:** Dietary intake at baseline and 16 weeks of the two groups and intervention effect.

Nutrient	Intervention		Control		Intervention effect^a^	
	Baseline	16 weeks	Baseline	16 weeks	Estimate (95% CI)	*p* value
Energy intake						
kJ	9143 (2591)	8497 (2077)	9904 (3912)	9450 (4080)	−247.13 (−1634.65, 1140.39)	0.72
kcal	2185 (619)	2030 (496)	2367 (935)	2258 (975)	−59.04 (−390.50, 272.43)	
Carbohydrate						
g	222.3 (89.1)	125.3 (54.0)	228.9 (99.1)	220.3 (110.7)	−81.79 (−127.16, −36.42)	<0.0005
E%	40.7	24.7	38.7	39		
Protein						
g	106.2 (40.7)	119.2 (57.0)	104.5 (42.0)	105.0 (50.7)	12.59 (−10.40, 35.57)	0.28
E%	19.5	23.5	17.7	18.6		
Total fat						
g	88.5 (34.1)	107.7 (39.1)	102.4 (54.0)	96.5 (48.5)	21.72 (1.04, 42.40)	0.040
E%	36.5	47.8	39	38.5		
Saturated fat						
g	32.8 (13.3)	34.1 (14.3)	37.7 (19.7)	38.0 (20.2)	0.40 (−8.57, 9.36)	0.93
E%	13.5	15.1	14.3	15.2		
Monounsaturated fat						
g	33.2 (16.0)	41.0 (18.4)	38.1 (23.1)	34.9 (20.3)	10.33 (0.77, 19.88)	0.034
E%	13.7	18.2	14.5	13.9		
Polyunsaturated fat						
g	13.2 (9.7)	19.6 (11.4)	15.7 (13.0)	12.6 (7.9)	8.36 (2.83, 13.89)	0.003
E%	5.4	8.7	6	5		
Dietary fibre						
g	28.0 (11.3)	29.1 (10.4)	24.6 (11.8)	25.8 (13.9)	−0.70 (−6.26, 4.87)	0.81

Data are presented as mean (standard deviation). Data collected via 24-h recall and analysed using FoodWorks.

*E%* energy percentage, *CI* confidence interval, *g* grams, *kcal* kilocalories, *kJ* kilojoules.

^a^Between-group differential change from baseline to week 16 (intervention versus control) estimated through two-way interaction between group allocation and measurement time point.

Self-efficacy, as measured by the DMSES, increased in the intervention group by a mean 6.95 (SD 24.55) points and decreased in the control group by a mean 1.11 (SD 26.42) points, though the differential change was not significant between groups 8.18 (95% CI: −2.55 to 18.90; *p* = 0.14). There were no differences in diabetes-related comorbidities between groups. In terms of adverse effects of carbohydrate reduction, one intervention group participant reported several days of constipation when starting the LCD; one reported one instance of mild hypoglycaemia. Both cases were self-managed and reported after their occurrence. Eighty per cent of intervention group participants adhered to the intervention as advised—detailed usage and engagement outcomes were reported elsewhere [37].

## Discussion

This study was the first to assess the effect of a LCD (10–<26% total energy intake from carbohydrate) provided in a web-based setting for people with T2DM using an RCT design. In this study, the web-based T2Diet programme provided in conjunction with standard care resulted in significant reductions in HbA1c, weight, BMI, and anti-glycaemic medication requirements compared with standard care alone. Our findings are consistent with previous meta-analyses of face-to-face LCD interventions in people with T2DM, where reductions in HbA1c, weight, BMI, and medication requirements have been repeatedly noted [6–9]. For the first time, this study provided evidence that these beneficial clinical outcomes can be achieved through a web-based setting to provide remote dietary support for people with T2DM in conjunction with standard care, which was significantly more effective than providing patients with standard care alone. These findings are important as improving access and availability to dietary education to support improved health in T2DM is urgently needed [21]; and currently there is a paucity of empirical evidence regarding web-based dietary interventions for people with T2DM [30].

The results supported our hypothesis that the intervention group would achieve lower HbA1c at 16 weeks, with a mean between-group difference of −0.65%, which is a clinically meaningful reduction [2]. An estimated 12% of diabetes-related deaths could be prevented with a modest 0.1% HbA1c reduction [38]. A decrease in HbA1c of 0.5% may avert cardiovascular events by 10% over 5 years [39]. Appreciably, the mean within-group HbA1c change for the intervention group was almost 1% (−0.94%). Each 1% reduction in HbA1c represents a risk reduction of 21% for diabetes-related deaths, 14% for heart attacks, and 37% for microvascular complications [40].

Without dietary or lifestyle modification, the primary method to assist patients to attain glycaemic control is through prescription of anti-glycaemic medication [2, 41]. In the current study, the control group increased anti-glycaemic medication requirements, while the intervention group decreased medication requirements, with 25% of intervention group participants achieving a reduction of at least 20% at 16 weeks. Medication reduction is a marker of improved glycaemic control in itself, and where medication reductions occur, the intervention effect is likely underestimated [2, 14]. Effectively measuring and observing medication reduction in the context of a web-based dietary programme is an important outcome, which has not been adequately assessed in previous web-based dietary interventions in people with T2DM [30, 42].

In the current study, the intervention was not specifically designed to achieve weight loss, as intervention group participants were able to consume foods ad libitum. The overall mean daily energy intake was consistent with the estimated daily energy intake recommended for the demographic in this study [43]. Even so, participants in the intervention group lost substantially more body weight. In addition, 38% of intervention group participants achieved at least 5% weight loss. Weight loss of at least 5% is a recommended clinical guideline for T2DM patients with overweight or obesity [2, 41], as it can be a facilitator for reductions in HbA1c and medications [2, 44]. The weight loss could be explained by hormonal changes related to insulin secretion and action [45, 46] and/or improved overall metabolic function [44, 46].

In terms of dietary intake, participants in the intervention group consumed 24.7% total energy intake from carbohydrates, indicating acceptable adherence to the recommended LCD (10–<26%). In previous reviews of LCD RCTs, comparative nutritional breakdowns have indicated significant reductions in dietary fibre and increases in saturated fat [6], which has raised concerns about the nutritional quality of LCDs [14]. The current study has demonstrated that in the context of a healthy LCD intervention these can remain unaffected.

Although self-efficacy did increase in the intervention group and decrease in the control group, the intervention effects observed did not appear to be mediated by changes in self-efficacy but could have been brought about by other determinants not examined, such as increased knowledge, motivation, and/or intentions.

Several limitations of this study were previously noted in our published protocol [31]. In brief, we acknowledged the limitations of not collecting additional biomarkers related to cardiometabolic risk, physical activity, psychological wellbeing, hunger, and satiety. We also acknowledged lack of longer-term follow-up, which was not feasible for this study. Long-term studies and sustainability of dietary interventions are lacking in general, not exclusive to LCDs [14]. However, this study presents an important first step in evaluating web-based delivery of a LCD education programme in conjunction with standard care, where further research can be undertaken. For this study, anthropometric data were self-reported. Validity studies in Australian adults suggest that while participants may slightly over-report height and underreport weight, the discrepancies are small when compared with clinically measured values [47]. Measurement error and reporting bias are potential limitations of self-reported dietary measures in the context of any dietary intervention. Strengths of the study include the RCT design, community-based recruitment, well-matched groups, concealed allocation, blinding, and robust data management [31] to reduce bias and strengthen the quality and validity of the findings. In addition, a major strength was the ability to conduct the study remotely, reaching participants from wide geographical locations to support improved dietary self-management and health outcomes, even in the midst of the Covid-19 pandemic. In terms of generalisability, the study sample was recruited from the general community and, like the broader T2DM population, was sociodemographically diverse. This indicates web-based T2DM dietary self-management education is potentially suitability across a wide demographic—all genders, duration of diabetes, relationship status, varying levels of education, and for the older age group most frequently diagnosed with T2DM. Thus, the outcomes of this work pave the way for similar interventions across varying locations and contexts.

## Conclusion

The results of the T2Diet Study illustrate that an automated web-based healthy LCD education programme can be provided in conjunction with standard care to support adults with T2DM to achieve significant improvements in glycaemic control and reductions in body weight, BMI, and anti-glycaemic medication requirements. Future research will explore implementation among a larger number of participants and including a longer follow-up time. Implementation through primary care recommendations would also be valuable, since routine monitoring of T2DM is the primary role of the GP.

## Data Availability

Upon reasonable request, an ethically compliant, deidentified data set may be made available subject to appropriate ethical approvals. Please contact SMSI at shariful.islam@deakin.edu.au.

## References

[1] Evert AB, Dennison M, Gardner CD, Garvey WT, Lau KHK, MacLeod J (2019). Nutrition therapy for adults with diabetes or prediabetes: a consensus report. Diabetes Care.

[2] American Diabetes Association Professional Practice Committee. Standards of medical care in diabetes—2022. Diabetes Care. 2022;45:S17–38. 10.2337/dc22-SINT.

[3] American Diabetes Association. (2005). Standards of medical care in diabetes. Diabetes Care.

[4] Dening J, Islam SMS (2020). Defining a low carbohydrate diet: proposal for a standardized consensus of carbohydrate intake (Carb-Cal Model). Diabetes Res Clin Pract.

[5] Feinman RD, Pogozelski WK, Astrup A, Bernstein RK, Fine EJ, Westman EC (2015). Dietary carbohydrate restriction as the first approach in diabetes management: critical review and evidence base. Nutrition.

[6] Huntriss R, Campbell M, Bedwell C (2018). The interpretation and effect of a low-carbohydrate diet in the management of type 2 diabetes: a systematic review and meta-analysis of randomised controlled trials. Eur J Clin Nutr.

[7] Sainsbury E, Kizirian NV, Partridge SR, Gill T, Colagiuri S, Gibson AA (2018). Effect of dietary carbohydrate restriction on glycemic control in adults with diabetes: a systematic review and meta-analysis. Diabetes Res Clin Pract.

[8] Snorgaard O, Poulsen GM, Andersen HK, Astrup A (2017). Systematic review and meta-analysis of dietary carbohydrate restriction in patients with type 2 diabetes. BMJ Open Diabetes Res Care.

[9] McArdle PD, Greenfield SM, Rilstone SK, Narendran P, Haque MS, Gill PS (2019). Carbohydrate restriction for glycaemic control in type 2 diabetes: a systematic review and meta-analysis. Diabet Med.

[10] Goldenberg JZ, Day A, Brinkworth GD, Sato J, Yamada S, Jonsson T (2021). Efficacy and safety of low and very low carbohydrate diets for type 2 diabetes remission: systematic review and meta-analysis of published and unpublished randomized trial data. BMJ.

[11] Silverii GA, Botarelli L, Dicembrini I, Girolamo V, Santagiuliana F, Monami M (2020). Low-carbohydrate diets and type 2 diabetes treatment: a meta-analysis of randomized controlled trials. Acta Diabetol.

[12] Meng Y, Bai H, Wang S, Li Z, Wang Q, Chen L (2017). Efficacy of low carbohydrate diet for type 2 diabetes mellitus management: a systematic review and meta-analysis of randomized controlled trials. Diabetes Res Clin Pract.

[13] Schwingshackl L, Chaimani A, Hoffmann G, Schwedhelm C, Boeing H (2018). A network meta-analysis on the comparative efficacy of different dietary approaches on glycaemic control in patients with type 2 diabetes mellitus. Eur J Epidemiol.

[14] Wheatley SD, Deakin TA, Arjomandkhah NC, Hollinrake PB, Reeves TE (2021). Low carbohydrate dietary approaches for people with type 2 diabetes: a narrative review. Front Nutr.

[15] Davies MJ, Aroda VR, Collins BS, Gabbay RA, Green J, Maruthur NM (2022). Management of hyperglycemia in type 2 diabetes, 2022. A consensus report by the American Diabetes Association (ADA) and the European Association for the Study of Diabetes (EASD). Diabetes Care.

[16] Diabetes Australia. Position statement. Low carbohydrate eating for people with diabetes. 2018. https://www.diabetesaustralia.com.au/wp-content/uploads/Diabetes-Australia-Position-Statement-Low-Carb-Eating.pdf

[17] Dyson PA, Twenefour D, Breen C, Duncan A, Elvin E, Goff L (2018). Diabetes UK evidence-based nutrition guidelines for the prevention and management of diabetes. Diabet Med.

[18] British Dietetic Association. Low carbohydrate diets for the management of type 2 diabetes in adults. 2018. https://www.bda.uk.com/resource/low-carbohydrate-diets-for-the-management-of-type-2-diabetes-in-adults.html

[19] Scottish Intercollegiate Guidelines Network. Management of diabetes: a national clinical guideline. 2010. https://www.sign.ac.uk/assets/sign116.pdf

[20] American Diabetes Association. (2022). Facilitating behavior change and well-being to improve health outcomes: standards of medical care in diabetes—2022. Diabetes Care.

[21] Powers MA, Bardsley JK, Cypress M, Funnell MM, Harms D, Hess-Fischl A (2020). Diabetes self-management education and support in adults with type 2 diabetes: a consensus report of the American Diabetes Association, the Association of Diabetes Care & Education Specialists, the Academy of Nutrition and Dietetics, the American Academy of Family Physicians, the American Academy of PAs, the American Association of Nurse Practitioners, and the American Pharmacists Association. Diabetes Care.

[22] Australian Diabetes Educators Association. Workforce in diabetes education. 2019. https://members.adea.com.au/resources-2/workforce-in-diabetes-education/

[23] Odgers-Jewell K, Isenring EA, Thomas R, Reidlinger DP (2017). Group-based education for patients with type 2 diabetes: a survey of Australian dietitians. Aust J Prim Health.

[24] Siopis G, Jones A, Allman-Farinelli M (2020). The dietetic workforce distribution geographic atlas provides insight into the inequitable access for dietetic services for people with type 2 diabetes in Australia. Nutr Diet.

[25] National Diabetes Service Scheme. Living in rural and remote areas. 2021. https://www.ndss.com.au/living-with-diabetes/about-you/people-with-diabetes-living-in-rural-and-remote-areas/

[26] Kennedy M, Dunning T (2017). Diabetes education: essential but underfunded in Australia. Diabetes Prim Care Aust.

[27] Siopis G, Colagiuri S, Allman-Farinelli M (2021). Doctors identify regulatory barriers for their patients with type 2 diabetes to access the nutritional expertise of dietitians. Aust J Prim Health.

[28] Hemmati Maslakpak M, Razmara S, Niazkhani Z (2017). Effects of face-to-face and telephone-based family-oriented education on self-care behavior and patient outcomes in type 2 diabetes: a randomized controlled trial. J Diabetes Res.

[29] Mohseni M, Ahmadi S, Azami-Aghdash S, Mousavi Isfahani H, Moosavi A, Fardid M (2021). Challenges of routine diabetes care during COVID-19 era: a systematic search and narrative review. Prim Care Diabetes.

[30] Dening J, Islam SMS, George E, Maddison R (2020). Web-based interventions for dietary behavior in adults with type 2 diabetes: systematic review of randomized controlled trials. J Med Internet Res.

[31] Dening J, George ES, Ball K, Mohebbi M, Islam SMS (2022). Randomised controlled trial of a web-based low carbohydrate diet intervention for adults with type 2 diabetes: the T2Diet study protocol. BMJ Open.

[32] Garner D, Olmsted M, Bohr Y, Garfinkel P (1982). The eating attitudes test: psychometric features and clinical correlates. Psychol Med.

[33] Alexopoulos AS, Yancy WS, Edelman D, Coffman CJ, Jeffreys AS, Maciejewski ML (2021). Clinical associations of an updated medication effect score for measuring diabetes treatment intensity. Chronic Illn.

[34] McDowell J, Courtney M, Edwards H, Shortridge-Baggett L (2005). Validation of the Australian/English version of the Diabetes Management Self-Efficacy Scale. Int J Nurs Pract.

[35] Carpenter JR, Roger JH, Kenward MG (2013). Analysis of longitudinal trials with protocol deviation: a framework for relevant, accessible assumptions, and inference via multiple imputation. J Biopharm Stat.

[36] Cohen J. Statistical power analysis for the behavioral sciences. 2nd edn. New York: Lawrence Erlbaum Associates; 2008.

[37] Dening J, Zacharia K, Ball K, George ES, Islam SMS (2022). Exploring engagement with a web-based dietary intervention for adults with type 2 diabetes: a mixed methods evaluation of the T2Diet study. PLoS ONE.

[38] Khaw K, Wareham N, Luben R, Bingham S, Oakes S, Welch A (2001). Glycated haemoglobin, diabetes, and mortality in men in Norfolk cohort of European Prospective Investigation of Cancer and Nutrition (EPIC-Norfolk). BMJ.

[39] Heintjes E, Penning-van Beest FJA, Parasuraman SV, Grandy S, Pollack M, Herings RMC (2011). Population attributable risk (PAR) of macrovascular events associated with HbA1c, blood pressure or weight in patients with type 2 diabets mellitus: evidence from a dutch cohort. Value Health.

[40] Stratton IM, Adler AI, Neil AW, Matthews DR, Manley SE, Cull CA (2000). Association of glycaemia with macrovascular and microvascular complications of type 2 diabetes (UKPDS 35): prospective observational study. BMJ.

[41] The Royal Australian College of General Practitioners. Management of type 2 diabetes: a handbook for general practice. East Melbourne, VIC: RACGP; 2020.

[42] Saslow LR, Moskowitz JT, Mason AE, Daubenmier J, Liestenfeltz B, Missel AL (2020). Intervention enhancement strategies among adults with type 2 diabetes in a very low-carbohydrate web-based program: evaluating the impact with a randomized trial. JMIR Diabetes.

[43] U.S. Department of Health and Human Services, U.S. Department of Agriculture. Dietary guidelines for Americans. 2015-2020. 2015. http://health.gov/dietaryguidelines/2015/guidelines/

[44] Nicholas AP, Soto-Mota A, Lambert H, Collins AL (2021). Restricting carbohydrates and calories in the treatment of type 2 diabetes: a systematic review of the effectiveness of 'low-carbohydrate' interventions with differing energy levels. J Nutr Sci.

[45] Ludwig DS, Aronne LJ, Astrup A, de Cabo R, Cantley LC, Friedman MI (2021). The carbohydrate-insulin model: a physiological perspective on the obesity pandemic. Am J Clin Nutr.

[46] Taylor R (2008). Pathogenesis of type 2 diabetes: tracing the reverse route from cure to cause. Diabetologia.

[47] Pasalich M, Lee AH, Burke L, Jancey J, Howat P (2014). Accuracy of self-reported anthropometric measures in older Australian adults. Australas J Ageing.

